# Risk factors and outcome of hyperammonaemia in people with epilepsy

**DOI:** 10.1007/s00415-022-11304-7

**Published:** 2022-07-30

**Authors:** Angeliki Vakrinou, Elaine Murphy, Sanjay M. Sisodiya, Umesh Vivekananda, Simona Balestrini

**Affiliations:** 1 Department of Clinical and Experimental Epilepsy, UCL Queen Square Institute of Neurology, London, WC1N 3BG UK; 2 Chalfont Centre for Epilepsy, Gerrards Cross, SL9 0RJ UK; 3 Charles Dent Metabolic Unit, The National Hospital for Neurology and Neurosurgery, Queen Square, London, WC1N 3BG UK; 4 Neuroscience Department, Children’s Hospital Meyer-University of Florence, Florence, Italy

**Keywords:** Ammonia, Epilepsy, Antiseizure medications, Hyperammonaemia

## Abstract

**Background:**

Hyperammonaemia is a recognised complication of antiseizure treatment but risk factors leading to individual patient susceptibility and outcome remain unclear.

**Objective:**

To identify risk factors for hyperammonaemia and investigate the impact of its management on clinical outcomes.

**Methods:**

We carried out a retrospective observational study of adults with epilepsy who had ammonia tested over a 3-year period**.** Hyperammonaemia was defined as ammonia level > 35 μmol/L. Patients were classified into two groups: hyperammonaemic and non-hyperammonaemic. Association analyses and linear regression analysis were used to identify risk factors for hyperammonaemia.

**Results:**

We reviewed 1002 ammonia requests in total and identified 76 people with epilepsy who had ammonia concentration measured, including 26 with repeated measurements. 59/76 (78%) were found to have hyperammonaemia. There was borderline statistical significance of hyperammonaemia being less common in patients with an established monogenic/metabolic condition than in those with structural or cryptogenic epilepsy (*P* = 0.05). Drug resistance, exposure to stiripentol and oxcarbazepine were identified as risk factors for hyperammonaemia. We found a dose-dependent association between valproate and hyperammonaemia (*P* = 0.033). Clinical symptoms were reported in 22/59 (37%) of the hyperammonaemic group. Improved clinical outcomes with concurrent decrease in ammonia concentration were seen in 60% of patients following treatment adjustment.

**Conclusions:**

Drug resistance and exposure to stiripentol, oxcarbazepine or high-dose valproate are associated with an increased risk of hyperammonaemia. Clinicians should consider symptoms related to hyperammonaemia in patients on high-dose valproate or multiple antiseizure treatments. Prompt identification of hyperammonaemia and subsequent treatment adjustments can lead to improved clinical outcomes.

**Supplementary Information:**

The online version contains supplementary material available at 10.1007/s00415-022-11304-7.

## Introduction

Hyperammonaemia (HA) in the context of epilepsy is associated with several factors. In the acute setting, HA can be observed as a result of generalised seizures [1]. Rare inherited or metabolic conditions, such as urea cycle defects, can result in chronic hyperammonaemia [2]. However, the most common scenario is HA secondary to antiseizure medications (ASM), in particular valproate (VPA) with a highly variable prevalence of HA ranging from 2 to 80% [3–9]. Several studies have identified an association between HA and VPA therapy [6, 9–12], with a number of risk factors including age [9], VPA dose and blood concentration [5, 13], and concomitant use of VPA with liver enzyme inducers such as phenobarbital (PB), phenytoin (PHT) and carbamazepine (CBZ) [6, 14]. Emerging evidence also indicates an increased risk of HA in patients treated with VPA and carbonic anhydrase inhibitors (CAIs) such as topiramate (TPM) and acetazolamide (ACZ) [15–17]. Indeed Yamamoto et al*.* demonstrated that combined use of zonisamide (ZNS) and TPM was an important risk factor for increased ammonia concentration in children with epilepsy, irrespective of concurrent treatment with VPA [18]. However, beyond small case series, little is known about the risk of HA with the use of other ASMs.

The association between symptoms and ammonia concentration remains unclear, with a significant proportion of individuals with HA cases remaining apparently asymptomatic. Symptoms associated with HA include gastrointestinal upset, somnolence, aggression, fatigue, ataxia and exacerbation of seizures [19]. In rare cases, HA has been associated with severe, even fatal, encephalopathy [20]. Moreover, guidance on management of symptomatic and asymptomatic HA is variable. Murphy and Marquardt (1982) reported that patients with venous ammonia concentrations not exceeding 240 μg/dl (171 μmol/L), can be asymptomatic [3]. In contrast, Coulter and Allen (1981) recommended reducing the dose of VPA when the ammonia concentration exceeds 100 μg/dl (71 μmol/L) [10]. In a large-scale study conducted in adult epilepsy patients in Japan, HA was found in 43% of the cohort, with 40.7% of them presenting with symptomatic HA requiring treatment with carnitine rescue, intravenous therapy or VPA dose reduction [6]. However, little has been reported on the effect of reduction of the offending drug on both ammonia concentration and patient symptoms.

In this study, we retrospectively examined over 1000 ammonia samples processed at our hospital over a 3-year period to answer the following questions. Is HA significantly associated with specific epilepsy types and/or aetiologies? Secondly, is HA associated with newer ASMs (e.g. levetiracetam (LEV), oxcarbazepine (OXC))? Finally, how does ASM dose adjustment affect patient outcomes in terms of symptoms and ammonia concentration?

## Methods

This project was reviewed and registered as an observational service evaluation at the National Hospital for Neurology and Neurosurgery, University College London Hospitals NHS Foundation Trust, London (UK). Ethics committee approval or individual participant consent is not required for a service evaluation, as service evaluation and clinical audit by definition do not involve intervention beyond usual clinical management [21].

Adults (> 18 years) with an established diagnosis of epilepsy due to any aetiology, who had ammonia tested from February 2016 to February 2019 at the National Hospital for Neurology and Neurosurgery were included in this study, either during inpatient admission or outpatient visits. Clinical information was obtained retrospectively from medical records.

The following variables were included in the analysis: age, gender, type and aetiology of epilepsy [22], duration of epilepsy at the time of ammonia measurement, comorbidities, drug resistance [23], types and frequency of seizures at the time of testing, types, doses, and blood concentrations of ASMs used at the time of measurement, presence of abnormal hepatic or renal function at the time of measurement, clinical symptoms associated with HA, treatment adjustment after HA detection, clinical outcome at least six months after HA detection.

Drug resistance was defined as failure of adequate trials of two tolerated and appropriately chosen and used ASM schedules (whether as monotherapies or in combination) to achieve sustained seizure freedom, according to the consensus recommendation of the International League Against Epilepsy (ILAE). Seizure freedom was defined as no seizure attacks for at least 12 months under current ASM according to the consensus proposal of the International League Against Epilepsy (ILAE) [23].

The upper limit of the reference range for blood ammonia concentration at our centre is 35 μmol/L. We defined hyperammonaemia as a plasma ammonia concentration exceeding 35 μmol/L.

Hepatic dysfunction was classified as mild with transaminase values of 2–5 × upper limit normal (ULN), moderate 5–15 × ULN and severe > 15 × ULN [24]. Renal impairment was defined as mild with eGFR values of 60–89 mL/min, moderate eGFR 59–30 mL/min and severe with eGFR values < 30 mL/min [25].

### Statistical analyses

Data were analysed using Stata/IC 11.1 (StataCorp, College Station, TX). *P* value was considered significant when < 0.05*.* Data were tested for normal distribution. The significance of differences in clinical factors potentially associated with ammonia concentration was estimated by Pearson *χ*^2^ or two-sided Fisher’s exact test, as appropriate, for categorical variables, and by *t *test and Spearman’s Rho coefficient for continuous variables. To avoid alpha error inflation caused by multiple testing, *P* values were corrected with the Holm-Bonferroni correction method [26, 27]. To identify predictors of plasma ammonia concentration, we used multivariate linear regression analysis with ammonia as an outcome variable. Univariate associations with a *P* value < 0.20 were used to build the multivariate model.

## Results

**Fig. 1:**
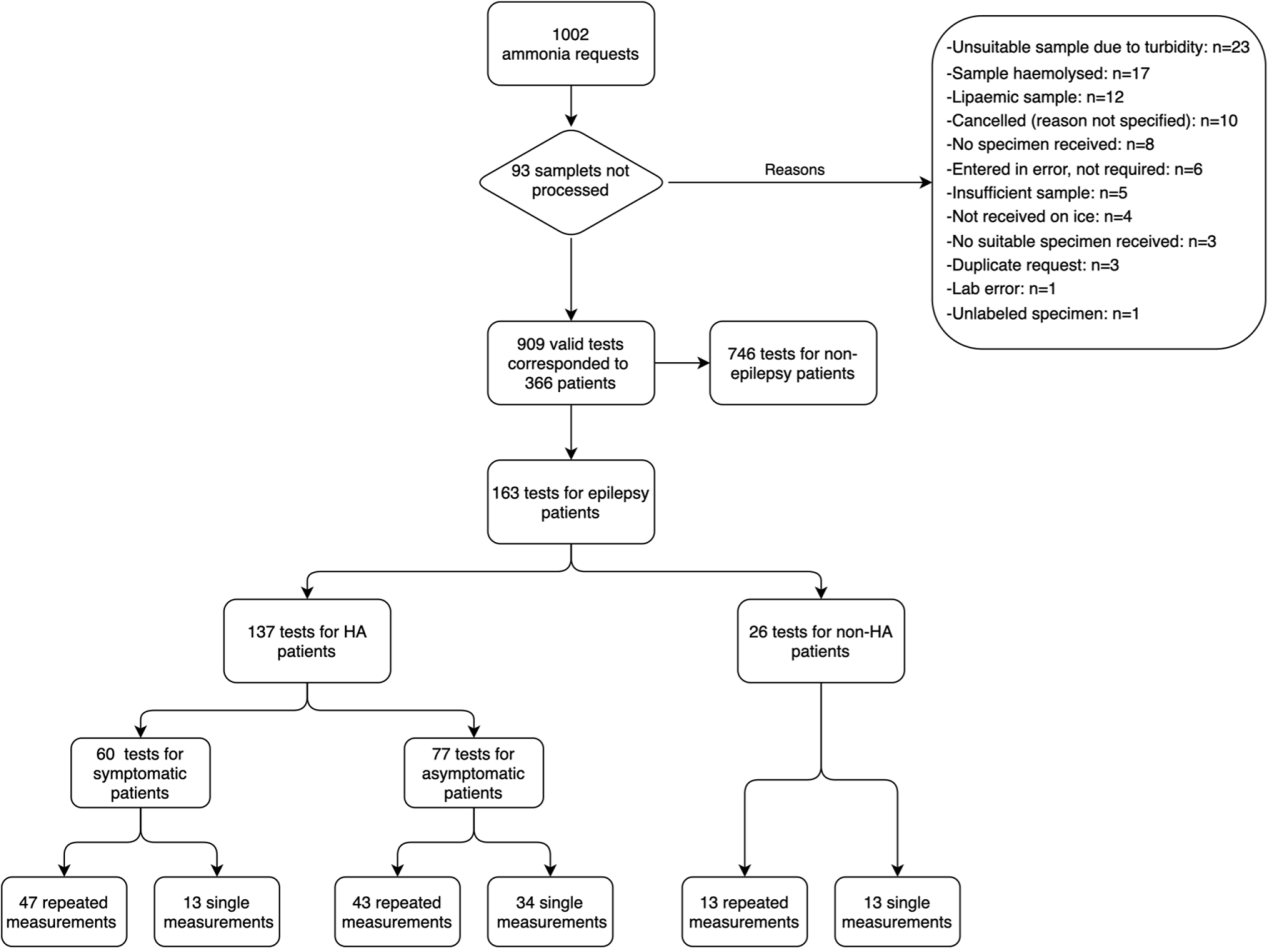
Ammonia measurements flowchart

Over the three  years’ interval considered, a total of 1002 ammonia tests were requested. Of these, 93 (9%) were not processed for various reasons, as illustrated in Fig. 1. The remaining 909 measurements were performed in 366 patients, 76 of which had a formal diagnosis of epilepsy. Of these 76, 17 had consistently normal ammonia concentrations (non-HA) whilst 59 had HA at least on one measurement (HA). Ammonia measurements were repeated on average 5 times (SD ± 2) in the HA group, and 1.5 times (SD ± 1) in the non-HA patients (*P* = 0.08) (Online Resource 1). Repeat measurements were conducted over a period of six to 24 months. The demographic data of HA and non-HA patients are summarised in Table 1.

**Table 1:** Demographic and clinical data of patients with detection of hyperammonaemia at least on one measurement (HA) and with consistently normal ammonia levels (non-HA)

	Hyperammonemia group	Non-Hyperammonemia group	*P* Value (uncorrected)
Number of patients	59	17	
Age (years)	41 (SD 17)	42 (SD 18)	0.97
Gender: female/male	23/36	9/8	0.40
Duration of epilepsy (years)	17 (SD 13)	18 (SD 18)	0.76
Epilepsy type; *N* (%)			
Focal	44 (75%)	11 (64%)	0.53
Generalized	13 (22%)	5 (29%)	0.53
Unknown	2 (3%)	1 (5%)	0.53
Epilepsy aetiology; *N* (%)			
Structural	17 (29%)	5 (29%)	> 0.99
Genetic^a^	9 (15%)	2 (11%)	> 0.99
Metabolic	5 (8%)	3 (17%)	0.36
Immune	1 (2%)	0	> 0.99
Infectious	1 (2%)	0	> 0.99
Unknown	26 (44%)	6 (35%)	0.28
Drug resistance; *N* (%)			
Drug-resistant	44 (75%)	9 (52%)	0.05
Drug responsive	15 (25%)	8 (47%)	0.13
Seizure control; *N* (%)			
Seizure free	13 (22%)	4 (23%)	> 0.99
Non-seizure free	46 (77%)	13 (76%)	> 0.99
Mode of ASM therapy; *N* (%)			
Monotherapy	10 (16%)	6 (35%)	0.17
Polytherapy	49 (83%)	11 (64%)	0.17
VPA therapy; *N* (%)			
VPA monotherapy	6 (10%)	3 (16%)	0.4
VPA combination therapy	39 (66%)	12(70%)	> 0.99
Concomitant medications; *N* (%)			
Antipsychotics	3 (5%)	0	> 0.99
Antidepressants	8 (13%)	2 (3%)	> 0.99
Lipid lowering agent	4 (7%)	1 (2%)	> 0.99
Antihypertensive agent	4 (7%)	2 (3%)	0.6
Orla hypoglycemic agent	1 (2%)	1 (2%)	0.39
Dose of VPA (mg/day)	1619 (SD 660)	975 (SD 508)	0.003^b^
Comorbidity; *N* (%)			
CNS	41 (44%)	7 (41%)	0.06
Metabolic	15 (16%)	4 (23%)	0.23
Other	35 (38%)	7 (41%)	0.26
None	1 (1%)	0	> 0.99
Not known	1 (1%)	2 (11%)	0.12
Established monogenic/metabolic condition; *N* (%)	5 (8%)	5 (29%)	0.01^c^
Plasma ammonia (μmol/L)	57 (SD 24)	26 (SD 5)	0.0001^b^

*SD* standard deviation; *ASM* antiseizure medication; *VPA* sodium valproate; *CNS* central nervous system

^a^Genetic aetiology includes people with molecularly confirmed monogenic conditions as well as people with idiopathic generalised epilepsies

^b^*P* value retained statistical significance following Holm-Bonferroni correction

^c^*P* value showed trend towards significance following Holm-Bonferroni correction

**Fig. 2:**
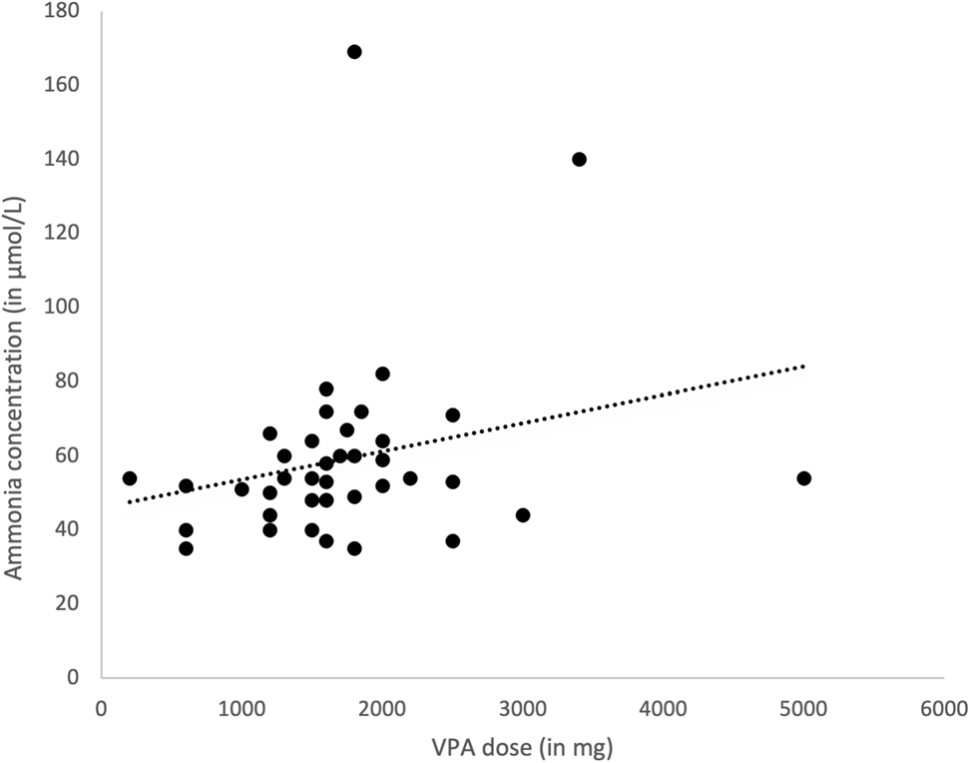
Correlation between ammonia concentration (in μmol/L) and sodium valproate (VPA) dosage (in mg)

Measurement of ASM levels was conducted in 31 patients (52%) of the HA group and in eight patients (47%) of the non-HA group. ASM levels above therapeutic range were found in six patients (10%) of the HA group; all eight patients of the non-HA group had ASM concentrations within therapeutic range (*P* = 0.31). As expected, VPA dosage was significantly higher in the HA group compared to the non-HA group (*P* = 0.033) (Fig. 2). However importantly, multivariate linear regression analysis identified as ammonia concentration predictors the following factors: drug-resistant epilepsy, exposure to OXC and stiripentol (STP) (Table 2), suggesting that clinical vigilance is needed when prescribing these ASMs. Details about the cases that were on either OXC or STP treatment are provided in Online Resource 2.

**Table 2:** Multivariate linear regression model using ammonia level as continuous outcome variable

	Coef.	Std. Err	*t*	*P* >|*t*|	[95% Conf. interval]	
Oxcarbazepine	29.380	10.180	2.89	0.005	9.074	49.685
Stiripentol	118.394	19.990	5.92	0	78.524	158.265
Drug-resistant epilepsy	10.940	5.049	2.17	0.034	0.869	21.011

Most of the subjects had normal liver and renal function at the time ammonia concentration was measured (88% in HA and 86% in non-HA groups) with the rest having only mild derangement.

There was a trend towards significance for HA being less common in patients with molecularly confirmed monogenic or metabolic conditions than in those with structural or cryptogenic epilepsy (*P* = 0.05): 5/59 individuals had a genetic or metabolic condition in the HA group (i.e. arginase deficiency, carnitine palmitoyltransferase I deficiency, Batten disease, *SCN1A*-related epilepsy and dentatorubral-pallidoluysian atrophy (DPRLA)) vs 5/17 in the non-HA group (i.e. ornithine transcarbamylase deficiency in two patients, arginosuccinic aciduria, Lennox-Gastaut syndrome and familial porencephaly associated with *COL4A1* pathogenic variant).

### Symptomatic patients

The mean ammonia concentration of all 59 HA patients was 57 ± 24 μmol/L (range 35–169 μmol/L) with ammonia concentration between symptomatic and asymptomatic patients approaching statistical significance (64.55 ± 34 μmol/L versus 53.5 ± 6 μmol/L, *P* = 0.057). Out of the 59 HA patients, 22 reported symptoms at the time of measurement. The most common symptoms included lethargy (*n* = 9), cognitive impairment (n = 7), irritability (n = 1), tremor (*n* = 4) and ataxia/unsteadiness (*n* = 1). In the non-HA group, one patient (1/17) reported symptoms of mild tremor and was on VPA monotherapy. Of the symptomatic patients, 19 (86%) were on polytherapy, with nine (40%) of them receiving more than three ASMs. The remaining three (13%) symptomatic patients were on monotherapy with VPA (Online Resource 3).

**Fig. 3:**
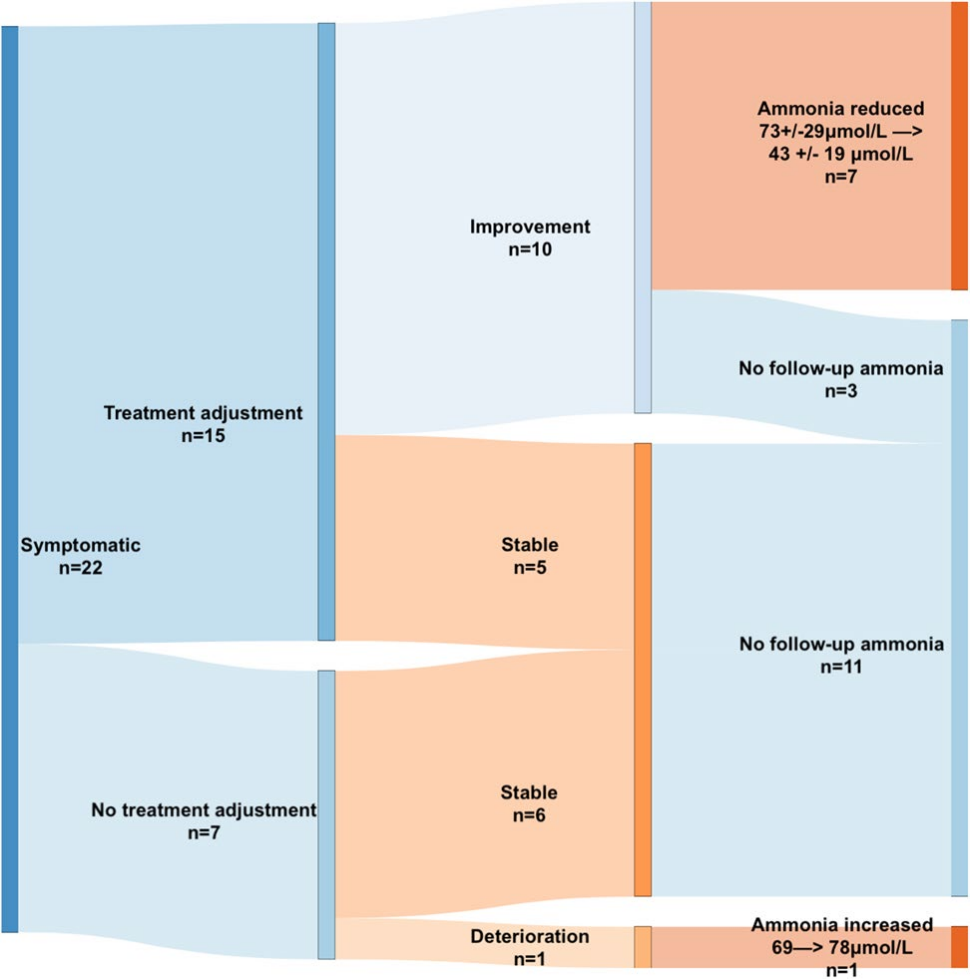
Symptomatic Patients—Clinical outcomes and follow-up ammonia measurements

Details of treatment adjustments with subsequent clinical outcomes are shown in Table 3. Of the seven patients who did not undergo medication changes after HA detection, six (85%) had stable symptoms and unchanged seizure control, whilst one patient (15%) deteriorated clinically with worsening lethargy and confusion but stable seizure frequency, at six-month follow-up. (Fig. 3).

**Table 3:** Treatment adjustment and clinical outcomes of HA patients

Patient no	Treatment prior to HA detection	Treatment adjustment after HA detection	Outcome
Symptomatic HA patients (*n* = 15)			
#1	VPA, LEV, PHT	VPA discontinued, LCS introduced	Improvement of cognitive impairment Stable seizures
#2	VPA, LTG	Reduction of VPA	Improvement of lethargy Stable seizures
#3	VPA, LCS, PGB	Reduction of LCS, introduction of CLB, VPA and PGB unchanged	Stable symptoms (fatigue) Stable seizures
#4	VPA, PHT	VPA reduction	Improvement of tremor Stable seizures
#5	VPA, OXC, CLB, BRV	VPA reduction, Increase in BRV	Improvement of lethargy Improvement of seizures
#6	VPA, PHT, OXC	VPA and PHT reduction	Improvement of ataxia Stable seizures
#7	VPA, OXC	OXC increase	Improvement of cognitive impairment Stable seizures
#8	VPA	Addition of LTG	Stable symptoms (lethargy and tremor) Improvement of seizures
#9	VPA, LEV	Increase in LEV, VPA unchanged	Improvement of cognitive impairment Improvement of seizures
#10	VPA, CBZ	Increase in CBZ, VPA unchanged	Stable symptoms (tremor) Improvement of seizures
#11	VPA, LTG, LEV, CLB	VPA reduction	Improvement of cognitive impairment Stable seizures
#12	VPA, STP, CLB	Reduction of VPA	Improvement of lethargy Improvement of seizures
#13	TPM, PB, CLB	Reduction of PB and CLB, TPM withdrawal Introduction of LCS	Improvement of cognitive impairment Improvement of seizures
#14	VPA, LTG, PER, CLB	Increase in VPA, reduction of LTG	Stable symptoms (tremor) Deterioration in seizures
#15	VPA, LTG	LTG withdrawal, introduction of LEV	Improvement of cognitive impairment Stable seizures
Asymptomatic HA patients (*n* = 21)			
#16	VPA, LEV, PHT	PHT reduction, VPA increase	Background of neurocytoma. Seizure worsening with functional decline and EEG features of encephalopathy
#17	LEV, VPA	VPA reduction	Seizure worsening New symptoms of upper limb tremor
#18	LEV, CBZ, CLB	LEV increase	Stable seizures New onset of behavioural difficulties
#19	LEV, LTG, PER, CZP	LEV reduction, PER increase	Seizure improvement
#20	VPA, CLB	VPA increase	Stable seizures
#21	TPM, PHT, CLB	LCS introduction	Stable seizures New symptoms of hair loss
#22	VPA, LTG	VPA increase	Stable seizures New onset of tremor
#23	VPA	VPA increase	Seizure improvement
#24	VPA, LEV, LZP	VPA reduction	Stable seizures
#25	LEV, PHT	PHT reduction	Seizure worsening
#26	VPA	VPA reduction	Stable seizures New onset of tremor
#27	VPA, LTG, LCS	LCS increase	Seizure worsening
#28	VPA, PHT, CBZ, LEV	VPA withdrawal, CLB introduction	Stable – seizure free
#29	VPA, LEV	VPA reduction	Stable seizures Improved cognition
#30	VPA, LEV	VPA increase	Seizure improvement
#31	VPA, LCS, ZNS	VPA and ZNS withdrawal	Stable–seizure free
#32	LEV, LCS	LEV increase, LCS withdrawal	Seizure improvement
#33	VPA, LTG	LTG reduction	Seizure improvement
#34	VPA, LTG	VPA increase, LTG withdrawal	Stable–seizure free
#35	ZNS	VPA introduction	Seizure improvement
#36	VPA, LEV, ZNS	ZNS increase	Seizure improvement

*HA* hyperammonaemia; *ASM* antiseizure medication; *EEG* electroencephalogram; *VPA* sodium valproate; *LEV* levetiracetam; *CBZ* carbamazepine; *PHT* phenytoin; *CLB* clobazam; *CZP* clonazepam; *TPM* topiramate; *LCS* lacosamide; *LTG* lamotrigine; *OXC* oxcarbazepine; *PGB* pregabalin; *STP* stiripentol; *PB* phenobarbital; *PER* perampanel; *BRV* brivaracetam; *LZP* lorazepam; *ZNS* zonisamide

### Asymptomatic patients

Mean ammonia concentration of the asymptomatic HA patients was 53.5 ± 6 μmol/L (range 36–127 μmol/L). Out of the 37 asymptomatic HA patients, 21 (56%) had changes in their ASM therapy (Table 4). The ASM changes in this asymptomatic group were not conducted in response to HA detection but rather as attempts to either improve seizure control (*n* = 18) or to withdraw ASM due to seizure remission (*n* = 3). Clinical outcomes following treatment changes are shown in Table 3. Of the asymptomatic patients who did not have ASM changes, 15 (82%) had stable seizure control at follow-up whilst one reported seizure worsening. All remained asymptomatic.

**Table 4:** Follow-up ammonia measurements in HA group

HA patients with serial ammonias	Baseline ammonia (μmol/L)	Ammonia at follow-up (μmol/L)	Symptoms	ASM changes
Symptomatic (*n* = 8)				
Treatment adjustment (*n* = 7)				
Patient #1	40	37	Improvement of cognitive impairment	On VPA/LEV/PHT. Withdrawal of VPA and introduction of LCS
Patient #2	67	37	Improvement of lethargy	On VPA/LTG. Reduction of VPA
Patient #6	140	81	Improvement of ataxia	On VPA/PHT/OXC. Reduction of VPA and PHT
Patient #7	69	48	Improvement of cognitive symptoms	On VPA/OXC. Increase of OXC
Patient #9	57	35	Improvement of cognitive impairment	On VPA/LEV. Increase of LEV
Patient #11	78	48	Improvement of cognitive impairment	On VPA/LTG/LEV/CLB. Reduction of VPA
Patient #15	59	17	Improvement of cognitive impairment	Withdrawal of LTG of a therapy with VPA/LTG and introduction of LEV
No treatment adjustment (*n* = 1)				
Patient #37	69	78	Deterioration of lethargy/confusion	On VPA/LTG/LEV/CLB
Asymptomatic (*n* = 7)				
Treatment adjustment (*n* = 3)				
Patient #21	75	56	Stable—asymptomatic	On PHT/CLB/TPM. Introduction of LCS
Patient #17	61	45	New symptoms of upper limb tremor	On VPA/LEV. VPA reduction
Patient #16	49	131	Background of neurocytoma. New clinical deterioration with functional decline and EEG features of encephalopathy	On VPA/LEV/PHT. PHT reduction initially, followed by LCS introduction. VPA reduction and increase in LCS when ammonia detected at 131(μmol/L)
No treatment adjustment (*n* = 4)				
Patient #38	108	127	Stable–asymptomatic	On LEV/CLB Background of OTC deficiency
Patient #39	46	68	Stable–asymptomatic	On CLB/LEV/LTG/LCS Background of OTC deficiency
Patient #40	54	43	Stable–asymptomatic	On TPM/LTG
Patient #41	67	46	Stable–asymptomatic	On OXC/CZP/LEV/PHT

*HA* hyperammonaemia; *ASM* antiseizure medication; *EEG* electroencephalogram; *VPA* sodium valproate; *LEV* levetiracetam; *PHT* phenytoin; *CLB* clobazam; *CZP* clonazepam; *TPM* topiramate; *LCS* lacosamide; *LTG* lamotrigine; *OXC* oxcarbazepine; *OTC* ornithine transcarbamylase

Table 4 shows HA patients with follow-up ammonia measurements. Follow-up monitoring was conducted in eight (36%) of the symptomatic patients and in seven (18%) of the asymptomatic patients. Of these, seven of the HA symptomatic patients who underwent ASM changes showed clinical improvement at follow-up with concurrent reduction of ammonia levels from an initial mean value of 73 ± 29 μmol/L down to 43 ± 19 μmol/L. The eighth HA patient had no ASM adjustment and showed clinical deterioration with consequent rise in ammonia levels at six-month follow-up. (Fig. 3). Variability in outcomes and follow-up ammonia measurements was seen in the asymptomatic HA group, with or without medication adjustments (Table 4).

## Discussion

In this retrospective study, we investigated a single-centre cohort of adult individuals with epilepsy who had ammonia measured, to identify risk factors for HA and investigate the impact of its management on clinical outcomes.

Our study identified drug-resistant epilepsy as a risk factor for HA. About one-third of people with epilepsy have seizures that do not effectively respond to pharmacological treatments [28]. Although the pathophysiological mechanisms underpinning drug resistance are not fully understood, it is well-established that people with drug-resistant epilepsy bear a high risk of comorbidities, injuries and mortality [29]. On this basis, it becomes crucial to rationalise treatment and reduce risk of side effects and additional symptoms related to ineffective therapies.

Surprisingly, there was a trend towards significance for HA being less common in individuals with a molecularly confirmed monogenic or metabolic condition than patients with other epilepsy aetiologies. The majority of the identified metabolic conditions in our cohort were urea cycle disorders, which are typically associated with elevated plasma ammonia concentrations [30]. A possible reason is that this category of patients is usually followed-up in highly specialised genetic or metabolic clinics with closer clinical vigilance, likely including regular ammonia monitoring and avoidance of medication known to cause increased HA risk (e.g., in our cohort none of the individuals with urea cycle disorders were on VPA treatment). These patients are also often treated with ammonia chelation and a restricted protein diet, which will also lower ammonia. However, it does suggest that ASM treatment should always be considered as a potential cause for HA irrespective of the type and aetiology of epilepsy.

Multiple-risk factors for HA have been identified in people treated with VPA including age, gender, VPA dose and combination therapy with enzyme inducers or carbonic anhydrase inhibitors. It is well documented that the ammonia increase induced by VPA is dose-dependent [6, 7, 9, 18]. We further confirm this in our study where we found that the concentration of ammonia was significantly correlated with the dosage of VPA. Several mechanisms have been described that may contribute to VPA-induced hyperammonaemia. VPA is metabolised in the mitochondria to valproyl-coenzyme A, which inhibits N-acetylglutamate synthase, thereby limiting detoxification of ammonia to carbamoyl phosphate and also resulting in secondary carnitine deficiency [19]. VPA also increases renal ammonia production by enhancing glutamine uptake in the renal mitochondria. Glutamine is subsequently converted to glutamate and ammonia in the renal tubules [31].

Apart from the well-known association of HA with VPA therapy, our data showed a potential association of HA with other ASMs, such as STP and OXC. HA is not reported in the summary of product characteristics of either drug. Recently, HA was detected in adult patients with Dravet syndrome who received STP for the first time in addition to VPA and clobazam (CLB): 77% of the small cohort of 13 patients were found to have hyperammonaemic encephalopathy after introduction of STP despite concomitant reduction of VPA and CLB doses. Symptoms as well as ammonia concentration improved after administration of carnitine allowing STP treatment to continue [32]. In our study, one patient had a diagnosis of Dravet syndrome and was on STP treatment in combination with VPA and CLB. They had severe hyperammonaemia with ongoing symptoms of drowsiness. Reduction of VPA led to improvement of drowsiness without seizure aggravation. One previous case report described hyperammonaemic encephalopathy after addition of PB to longstanding TPM and OXC therapy [33]. Four patients in our cohort were on OXC treatment, all on combination therapy, with reported symptoms of lethargy (*n* = 1), ataxia (*n* = 1), cognitive difficulties (*n* = 1), or asymptomatic (*n* = 1). Three of them were on both OXC and VPA, and in two reduction of concomitant VPA, with additional PHT reduction in one, led to improvement of lethargy (*n* = 1) and ataxia (*n* = 1). Interestingly, the third patient who was on OXC and VPA treatment, had reported improvement of cognitive difficulties following OXC increase, with stable mildly increased ammonia levels. The fourth patient, with asymptomatic HA, was on OXC in combination with LEV and PHT and clonazepam (CLZ). No medication changes were made and at follow-up they remained asymptomatic with slightly reduced ammonia levels (Online Resource 2). Given the limited power of our study due to small sample size, inferences about the strength of association between HA and STP or OXC cannot be made.

In the current study, HA was found in 55% of the patients on VPA monotherapy and in 79% of the patients with VPA combination therapy. Importantly, for the patients on ASM therapy other than VPA, raised ammonia levels were found in 60% (9/15) of those on polytherapy compared to 14% (1/7) on those in monotherapy. Although not statistically significant in our study, it has been previously noted by Yamamoto et al. that ammonia concentration showed a significant increase with a higher number of ASMs concomitant to VPA [6].

Our study demonstrated that HA can often be an asymptomatic finding in patients with epilepsy but occasionally may be associated with symptoms. It has been reported in a few studies that HA, with ammonia concentration > 56 μmol/l, can lead to encephalopathy and status epilepticus [34, 35], whilst in most people ASM-induced HA is of minor clinical significance. In two large-scale HA studies by Yamamoto et al., one in an adult and one in a paediatric cohort, symptoms were reported in 40.7% and 22.3% of the patients, respectively [6, 18]. In the adult cohort, patients with ammonia concentrations exceeding 200 μg/dL (143 μmol/L), reported increased seizure frequency or required treatment with carnitine rescue or VPA dose reduction. Similar issues were reported in the paediatric cohort with ammonia levels greater than 150 μg/dL (107 μmol/L). In our cohort, 37% of patients reported symptoms at the time HA was detected. Ammonia levels were only mildly raised and showed a trend towards statistically significant difference from the asymptomatic group. Why some individuals exhibit, or do not exhibit symptoms with similar plasma ammonia concentrations is still unclear. To the best of our knowledge, there are no studies investigating a potential genetic predisposition for HA intolerance, irrespective of plasma ammonia concentration.

Our cohort is highly heterogenous in terms of patient profiles, type and aetiology of epilepsy. Moreover, as most of the patients were on multiple ASMs it is difficult to delineate whether the reported symptomatology was secondary to either HA or polytherapy. Nevertheless, ammonia level emerged as possible contributing factor to adverse events reported during clinical follow-up. Of all patients who reported symptoms in our cohort, 73% (11/15) demonstrated clinical improvement following treatment adjustment. The changes in medications varied significantly within the symptomatic group which could support the contributory role of HA in the reported symptomatology. Contrastingly, unchanged symptomatology or clinical deterioration was observed in the patients whose treatment regimen remained unchanged after HA detection. Although we cannot prove causality, this suggests that ASM tailoring following identification of HA-related clinical symptoms may be associated with improvement in clinical outcomes.

Medication adjustment that was implemented in our study included dose reduction or discontinuation of VPA (6/11), reduction of PHT (2/11) and dose increase in other ASMs (LEV, LCS, OXC) (3/11). Similarly, Tseng et al. reported rapid recovery in all the patients with HA who were on VPA monotherapy or combination therapy and presented with symptoms, after correction of blood ammonia with adjustment in the dose of VPA or lactulose treatment [9].

Although there is no current recommendation for routine monitoring of ammonia in patients on VPA or other ASM therapy [36], a recent study by Meijer et al. recommended ammonia measurement in patients on treatment with VPA alone or combined with TPM who present with symptoms of encephalopathy, seizure worsening, behavioural changes, or gastrointestinal adverse effects such as nausea and vomiting. Reduction or discontinuation of VPA along with dietary protein intake monitoring are suggested as initial therapeutic approaches. Carnitine supplementation if carnitine deficiency is detected, and addition of nitrogen scavenger medications, have been recommended as additional considerations within the therapeutic strategy [31].

We could not systematically assess why measurement of ammonia was requested, given the retrospective nature of the study. Common reasons extrapolated from review of the medical records included ongoing treatment with VPA and reported symptoms suggestive of HA, given that the majority of the patients were on treatment with VPA (66% of both HA and non-HA groups) and 37% reported symptoms at the time of testing. In other cases, ammonia was tested as part of the routine set of blood testing in epilepsy patients. The lack of clear indication for requesting ammonia testing in epilepsy patients led us to conduct this service evaluation. Our results show that, when measured, ammonia levels and potential symptoms associated with HA can play an important role in optimising treatment strategies and subsequently improving clinical outcomes.

Our study has limitations that need to be acknowledged including the retrospective study design and the relatively small sample size despite screening an initial cohort of over 1000 samples sent for ammonia testing. Additionally, the accuracy of the blood ammonia values can be affected by pre-analytical conditions such as fist clenching, tourniquet use, and placement of the sample on ice [37]. Furthermore, blood ammonia levels could be influenced by factors related to patient’s profile such as fasting or time interval from the last convulsive seizure prior to measurement [38]. Due to the retrospective design of our study such parameters could not be controlled. In our cohort, 9% of blood samples could not be analysed due to the above or other reasons (Fig. 1). Moreover, it is difficult to confidently indicate whether patients were genuinely symptomatic from the retrospective review of the medical records. Additional parameters such seizure frequency and clinical outcomes could not be evaluated using formal measurements due to the retrospective data collection. Lastly, ammonia testing was carried out in a single specialist tertiary centre which may represent a less representative population of patients with epilepsy seen in non-specialist settings; although our results may provide guidance to ammonia testing in epilepsy which may prove useful also in primary and secondary care.

In conclusion, our study established some factors potentially associated with a higher risk of HA including drug resistance and exposure to STP or OXC, in addition to high-dose VPA. Additionally, identification of symptoms related to HA and subsequent adjustment of ASM regimen was associated with improved clinical outcomes for patients with detected HA. We suggest that ammonia should be monitored in people on high doses of VPA or on ASM polytherapy with or without VPA when patients report possible HA-related symptoms. Detection of HA and presence of HA-related symptoms should prompt consideration of treatment adjustment. Our findings provide guidance to minimise HA-related adverse effects and overall improve outcome in people with severe epilepsies.

## Supplementary Information

Below is the link to the electronic supplementary material.

Supplementary file1 (DOCX 5449 kb)

Supplementary file2 (DOCX 17 kb)

Supplementary file3 (DOCX 15 kb)
