# Supplementary material for: Risk factors and outcome of hyperammonaemia in people with epilepsy
Source: J Neurol. 2022 Jul 30;269(12):6395–405. doi: 10.1007/s00415-022-11304-7 (PMC9618503; doi:10.1007/s00415-022-11304-7)
Supplement: Supplementary file 2 — Supplementary file2 (DOCX 17 kb) [file 415_2022_11304_MOESM2_ESM.docx]

**Title:**

RISK FACTORS AND OUTCOME OF HYPERAMMONAEMIA IN PEOPLE WITH EPILEPSY

**Journal:** Journal of Neurology

**Authors:**

Angeliki Vakrinou^1,2^, Elaine Murphy^3^, Sanjay M Sisodiya^1,2^, Umesh Vivekananda^1††^, Simona Balestrini^1,2,4††^

**Affiliations:**

^1^Department of Clinical and Experimental Epilepsy, UCL Queen Square Institute of Neurology, London WC1N 3BG, UK

^2^Chalfont Centre for Epilepsy, Gerrard Cross SL9 0RJ, UK

^3^Charles Dent Metabolic Unit, The National Hospital for Neurology and Neurosurgery, Queen Square, London, WC1N 3BG, UK

^4^Neurology Unit and Neurogenetics Laboratories, Meyer Children Hospital, Florence, Italy

††Joint senior authors

**Correspondence to:**

Dr Simona Balestrini MD PhD

Department of Clinical and Experimental Epilepsy, UCL Queen Square Institute of Neurology, London WC1N 3BG, UK

Chalfont Centre for Epilepsy, Gerrard Cross SL9 0RJ, UK

Neurology Unit and Neurogenetics Laboratories, Meyer Children Hospital, Florence, Italy

Address: 33 Queen Square, London, WC1N 3BG

[s.balestrini@ucl.ac.uk](mailto:s.balestrini@ucl.ac.uk)

Online Resource 2

***Online Resource 2:*** *Treatment information for patients who were on oxcarbazepine (OXC) or stiripentol (STP) treatments*

Four patients were on OXC combination therapy.

Patient #5: He was a 37year-old gentleman with a diagnosis of refractory focal epilepsy of unknown aetiology. He had history of depression, for which he did not receive any pharmacological treatment. He was on treatment with sodium valproate (VPA), OXC, brivaracetam (BRV) and clobazam (CLB). His ammonia level was 54 μmol/L and he reported symptoms of lethargy. Treatment adjustment included VPA reduction and BRV increase, which led to improvement of lethargy and improved seizure control. No follow-up ammonia measurement was available.

Patient #6: She was a 64year-old lady with a diagnosis of drug-resistant focal epilepsy of unknown aetiology. She had history of depression, hypothyroidism and previous alcohol excess. She was on polytherapy with VPA, phenytoin (PHT) and OXC. In addition, she was on treatment with levothyroxine and citalopram as well as vitamin D and folic acid supplements. Ammonia level was 140 μmol/L and she reported symptoms of ataxia. Reduction of VPA and PHT led to marked improved of ataxia. Her seizure control remained unchanged. At follow-up, ammonia was reduced to 81 μmol/L.

Patient #7: He was a 19year-old gentleman with a diagnosis of medically refractory focal epilepsy. His medical history as remarkable for left temporal ganglioglioma which was resected at the age of 12 years. His ammonia level was 48 and he reported symptoms of cognitive impairment. An increase in OXC led to improvement of his cognitive difficulties. His seizure control remained unchanged. Follow-up ammonia level was unchanged at 48 μmol/L.

Patient #41: He was a 54year-old gentleman with focal epilepsy secondary to right focal cortical dysplasia, for which he underwent lesionectomy at the age of 48 years. He also had a background of psychosis and dissociative seizures. He was treated with OXC, PHT, levetiracetam (LEV) and clonazepam (CLZ) for his epilepsy. He was seizure free on these medications following surgery. He was also on treatment with aripiprazole, zopiclone and vitamin D supplements. His ammonia level was 61 μmol/L and he did not report any symptoms. No treatment adjustment was done, and he remained seizure free and asymptomatic at follow-up. He remained hyperammonaemic at follow-up with ammonia level of 46 μmol/L.

One patient on STP combination therapy:

Patient #12: He was a 21year-old gentleman with a diagnosis of severe myoclonic epilepsy of infancy secondary to a *de novo* mutation. His seizures were refractory to pharmacological treatment. He was on treatment with VPA, STP and clobazam (CLB). His ammonia level was 169 μmol/L with reported excessive drowsiness and lethargy. Reduction in VPA led to improvement of lethargy and improved seizure control. No further ammonia measurement was available at follow-up.
