# Supplementary material for: Risk factors and outcome of hyperammonaemia in people with epilepsy
Source: J Neurol. 2022 Jul 30;269(12):6395–405. doi: 10.1007/s00415-022-11304-7 (PMC9618503; doi:10.1007/s00415-022-11304-7)
Supplement: Supplementary file 3 — Supplementary file3 (DOCX 15 kb) [file 415_2022_11304_MOESM3_ESM.docx]

**Title:**

RISK FACTORS AND OUTCOME OF HYPERAMMONAEMIA IN PEOPLE WITH EPILEPSY

**Journal:** Journal of Neurology

**Authors:**

Angeliki Vakrinou^1,2^, Elaine Murphy^3^, Sanjay M Sisodiya^1,2^, Umesh Vivekananda^1††^, Simona Balestrini^1,2,4††^

**Affiliations:**

^1^Department of Clinical and Experimental Epilepsy, UCL Queen Square Institute of Neurology, London WC1N 3BG, UK

^2^Chalfont Centre for Epilepsy, Gerrard Cross SL9 0RJ, UK

^3^Charles Dent Metabolic Unit, The National Hospital for Neurology and Neurosurgery, Queen Square, London, WC1N 3BG, UK

^4^Neurology Unit and Neurogenetics Laboratories, Meyer Children Hospital, Florence, Italy

††Joint senior authors

**Correspondence to:**

Dr Simona Balestrini MD PhD

Department of Clinical and Experimental Epilepsy, UCL Queen Square Institute of Neurology, London WC1N 3BG, UK

Chalfont Centre for Epilepsy, Gerrard Cross SL9 0RJ, UK

Neurology Unit and Neurogenetics Laboratories, Meyer Children Hospital, Florence, Italy

Address: 33 Queen Square, London, WC1N 3BG

[s.balestrini@ucl.ac.uk](mailto:s.balestrini@ucl.ac.uk)

Online Resource 3:

| ***Online Resource 3.*** *Treatment regimen of symptomatic patients* | |
| --- | --- |
| Total number of symptomatic patients | 22 |
| VPA monotherapy | 3 |
| VPA combination therapy | 16 |
| Concomitant ASMs  Phenytoin (PHT)  Carbamazepine (CBZ)  Oxcarbazepine (OXC)  Lamotrigine (LTG)  Lacosamide (LCS)  Perampanel (PER)  Clobazam (CLB)  Clonazepam (CZP)  Stiripentol (STP)  Brivaracetam (BRV)  Pregabalin (PGB)  Levetiracetam (LEV) | 4  2  3  4  1  2  5  1  1  1  1  5 |
| Other combination therapy | 3 |
| Concomitant ASMs  Topiramate (TPM)  Phenobarbital (PB)  Clobazam (CLB)  Levetiracetam (LEV)  Lacosamide (LCS)  Pregabalin (PGB) | 1  1  2  2  1  1 |
| Abbreviations: VPA: sodium valproate; ASM: antiseizure medication | |
